# Influence of stromal elements on resident T cell migration in human and murine tumors analyzed by real-time imaging

**DOI:** 10.1186/2051-1426-3-S2-P390

**Published:** 2015-11-04

**Authors:** Elisa Peranzoni, Houcine Bougherara, Audrey Mansuet-Lupo, Vincent Feuillet, Julia Weiss, Fabienne Reigner, Diane Damotte, Marco Alifano, Marie-Aude Le Frere-Belda, Fabrice Lécuru, Emmanuel Donnadieu

**Affiliations:** 1 Institut Cochin INSERM U1016, Paris, France; 2 Department of Pathology, APHP, INSERM U1138, Paris, France; 3 Deparment of Thoracic Surgery, AP-HP, Paris, France; 4 Department of Pathology, Hôpital Européen Georges Pompidou, Université Paris Descartes, Paris, France; 5 Department of Gynaecological and Oncological Surgery, Hôpital Européen Georges Pompidou, AP-HP, Paris, France

## 

It is well established that T cells are crucial for the anti-tumor response, through the production of cytokines, direct cytotoxicity, and the activation of other “killer” cells of the innate arm. In order to perform their antitumor activities, T cells need to adequately respond to tumor antigens, be activated and be able to contact malignant cells. Up to now, our knowledge of how T cells migrate within tumors mainly comes from experiments performed in mouse models that do not fully recapitulate human cancers.

We have previously described a technique to track in real-time the motile behavior of fluorescent T cells plated onto fresh sections of human lung tumors. We have now refined this approach to monitor the locomotion of *resident* tumor-infiltrating CD8 T cells labeled with fluorescently-coupled antibodies. Using this approach our findings reveal that CD8 T cells accumulate in the stroma of ovarian and lung carcinomas but move slowly in this compartment. By contrast, even though less populated, tumors islets were found to be relatively good migration zones for CD8 T cells. As found in lung cancer, we have confirmed in ovarian tumors the key role played by collagen fibers of the extracellular matrix (ECM) which, by their orientation, spacing and density, control the distribution and migration of resident CD8 T cells within the tumor stroma.

In addition to ECM fibers, we have observed that CD8 T cells are frequently engaged in long-lived contacts with macrophages in the stroma of lung and ovarian carcinomas. Moreover, we have made similar observations in a different murine tumor models, both spontaneous and transplantable. The depletion of macrophages in these models causes an increased motility of CD8 T cells, suggesting that tumor-associated macrophages could induce a functional “paralysis” of CD8 lymphocytes that restricts their migration to the stromal compartment. However, macrophage depletion induces only a slight delay in tumor growth, suggesting that the restoration of T cell motility, and thus an augmented scanning ability of lymphocytes, is not sufficient to provoke tumor eradication. Given the importance of distinct components of the stroma in the reduction of T cell infiltration into tumor islets, we are now combining approaches that target macrophages or the ECM with checkpoint inhibitor immunotherapy, in order to improve and broaden its efficacy.

